# MED35/450: Evaluation of Internet Health Portals

**DOI:** 10.2196/jmir.1.suppl1.e72

**Published:** 1999-09-19

**Authors:** M Brucksch, C Lenz

**Affiliations:** ^1^Uni-Klinik Heidelberg, HeidelbergGermany

**Keywords:** Health Portal, Internet, Medical Education, Health Information

## Abstract

**Introduction:**

Currently, more than 22 million U.S. adults use the Internet to access health and medical information. More and more Health Care online portals - entry points for large numbers of online surfers looking for specialized news and information - try to satisfy the exploding demand.

**Methods:**

Using desk-research we have done an in-depth analysis of the emerging health portal market focusing on services offered, revenue strategies, typical online hurdles and threats, commerce and advertising potentials, and case studies of the main players.

**Results:**

We have identified several different groups of health portals and worked out the differences between the U.S. and German market concerning patient and physician services offered. Furthermore, we have evaluated the effectiveness of the Internet as a health and medical information provider compared with other media, such as televisions, newspapers and magazines.

**Discussion:**

Health portals will make patients better informed, more empowered to make health care choices, and, hopefully, healthier individuals.

